# Supplementary material for: Decoding the influence of anticipatory states on visual perception in the presence of temporal distractors
Source: Nat Commun. 2018 Apr 13;9:1449. doi: 10.1038/s41467-018-03960-z (PMC5899132; doi:10.1038/s41467-018-03960-z)
Supplement: Supplementary file 1 — Supplementary Information [file 41467_2018_3960_MOESM1_ESM.pdf]

SUPPLEMENTARY INFORMATION
(Supplementary Figures S1-S7)

Associated with the manuscript: ***Decoding the Influence of Anticipatory States on Visual Perception in the Presence of Temporal Distractors*** by Freek van Ede, Sammi R Chekroud, Mark G Stokes, and Anna C Nobre; as published in *Nature Communications*.

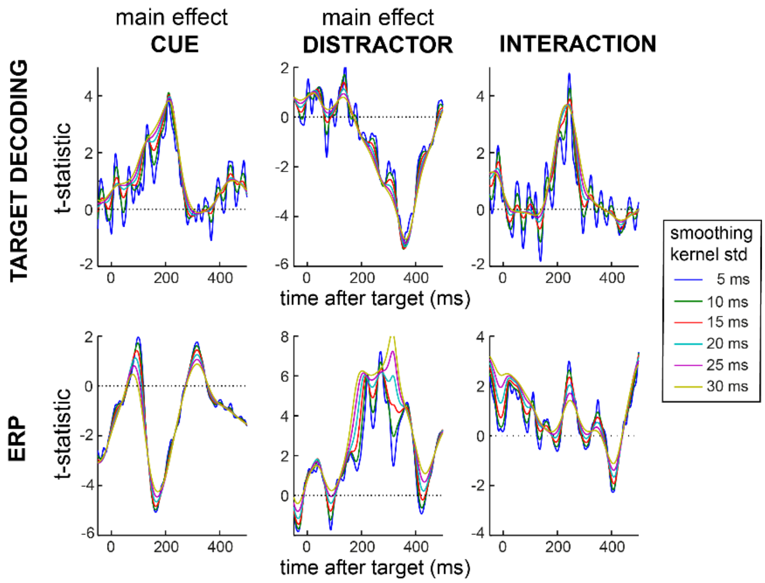

Supplementary Figure 1. Decoding and ERP effects are largely invariant to choice of smoothing kernel. Main effects of cue presence (left), distractor presence (middle), and their interaction (right) for both target decoding (upper) and ERPs (lower) as a function of the width (in standard deviations) of the applied Gaussian smoothing kernel (different colors). Plotted are the t-values associated with the simple contrasts (left: cued vs. uncued; middle: distractor present vs absent; right: cued minus uncued in distractor-present vs. distractor-absent trials; see also Figure 4c). All results reported in the manuscript were based on the smoothing kernel with the 15 ms standard deviation (i.e., the red line).

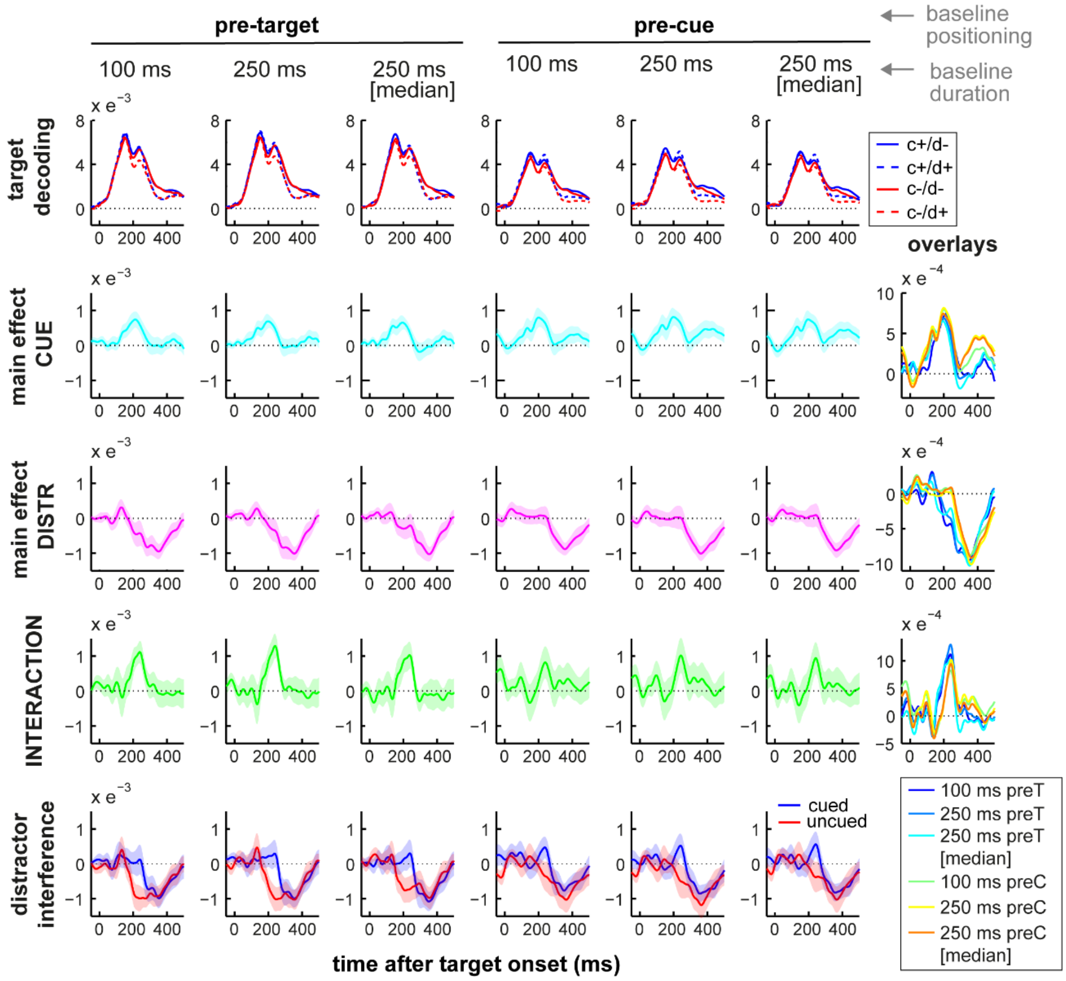

Supplementary Figure 2. Main decoding results for six different baseline variants. Baselines were either positioned pre-target or pre-cue and spanned either 100 or 250 ms. In addition to subtracting the mean EEG signal of each baseline, we also explored subtracting the median. All baselines were performed at the single-trial level and involved subtraction. The baseline used in all other analyses was the 250 ms pre-target baseline with mean subtraction (the second column). This baseline was chosen based on a-priori reasons – it is the closest to the target processing period of interest, whilst not containing any stimulus identity information –, and this baseline comparison was only made after all other reported analyses had already been evaluated. Shadings represent ± 1 s.e.m. calculated across participants (n = 30).

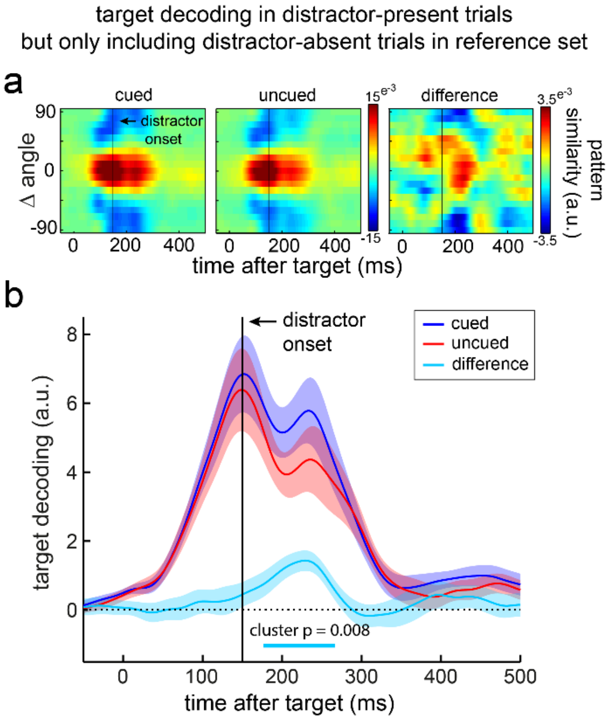

Supplementary Figure 3. Target decoding in distractor-present trials, when only including distractor-absent trials in the reference set. (**a**) Orientation decoding tuning profiles separately for cued and uncued trials, as well as their difference Reference sets were the same for cued and uncued trials; the reference set simply contained all distractor-absent trials, independent of cue-presence. (**b**) Corresponding time-resolved summary statistics of target decoding as a function of cue condition. Shadings represent ± 1 s.e.m. calculated across participants (n = 30).

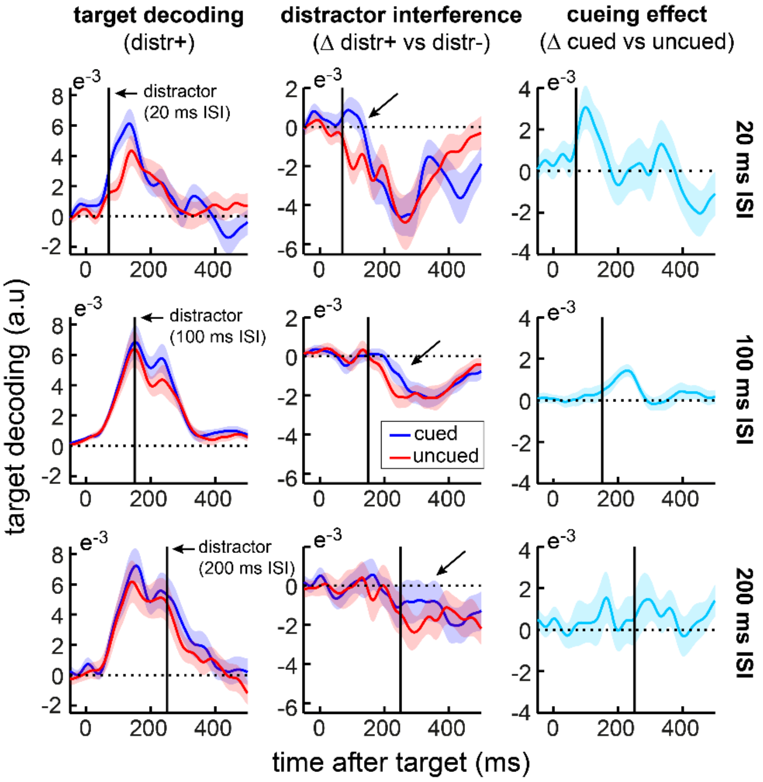

Supplementary Figure 4. Target decoding in cued and uncued distractor-present trials as a function of the interval between target and distractor. As in Supplementary Fig. 3, we only included distractor-absent trials in our reference sets to ensure a fair comparison between the 20, 100, and 200-ms ISI conditions. For the middle (distractor interference) panels, we subtracted the decoding in the distractor-absent trials (unshown) to yield the same type of distractor interference plot as in Figure 4b. Arrows indicate effects of interest, whereby distractor interference appears delayed in cued compared to uncued trials. The right panels show the difference between cued and uncued trials. Black vertical lines indicate the onset of the distractor in the different conditions. Shadings represent ± 1 s.e.m. calculated across participants (n = 30).

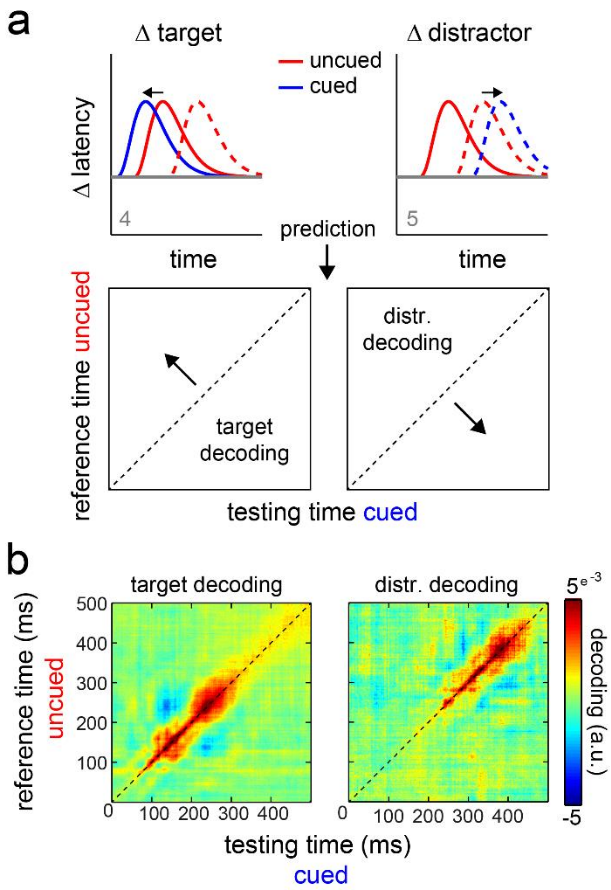

Supplementary Figure 5. Cross temporal decoding between cued and uncued trials reveals no cueing effect on the latencies of either target or distractor decoding. (a) Schematics of potential cueing effects on target (left) and distractor (right) decoding (taken from Fig. 3), together with predicted pattern in cross-temporal decoding analysis. If cueing results in an earlier target representation, then the cross-temporal plot (using uncued trials as the reference or "training" set and testing on cued trials) should reveal a leftward shift from diagonal when considering the decoding of the target orientation (e.g., the code at t = 100 during cued trials should resemble the code at t = 100+x in uncued trials). Similarly, if cueing results in delayed distractor coding, then this should reveal a rightward shift from the diagonal when considering the decoding of the distractor orientation. **(b)** Observed cross-temporal decoding data when only including uncued trials in the reference set and testing on cued trials. For both target and distractor decoding, these plots reveal a clear diagonal focus. Thus, while the "orientation code" is highly dynamic over time, the latencies of this dynamic code remain highly similar between cued and uncued trials.

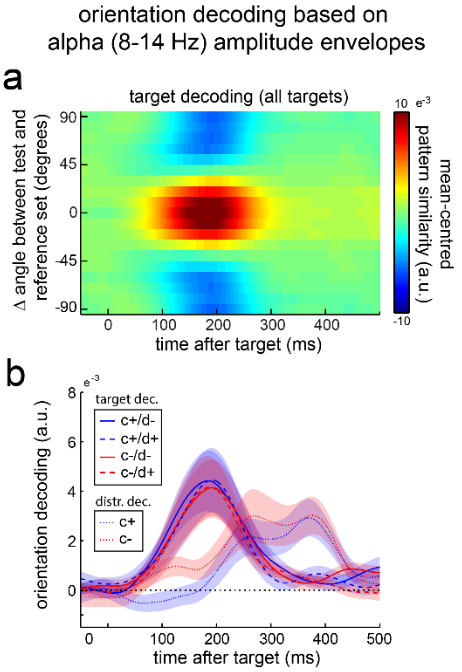

Supplementary Figure 6. Target and distractor decoding based on alpha amplitude envelopes. Conventions as in Figures 2a and 4a, except before running the decoding analysis, we band-pass filtered the time-domain signal in the 8-14 Hz alpha band and used the Hilbert transform to obtain time-varying amplitude envelopes. Shadings represent ± 1 s.e.m. calculated across participants (n = 30).

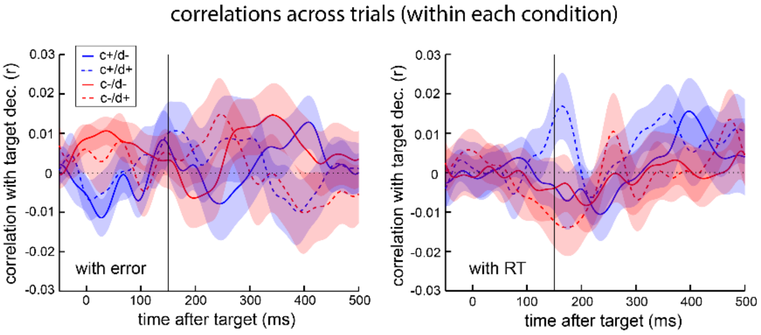

Supplementary Figure 7. Trial wise correlations between target decoding and behavioural performance. Time-resolved trial-wise correlations between target decoding and reproduction error (left) as well as reaction time (right), as a function of cue and distractor presence. Note that we hypothesised negative correlations, provided that better behavioural performance is associated with lower reproductions errors and lower RTs. The lines show the group-mean correlation values and the shadings represent ± 1 s.e.m. calculated across participants (n = 30).
